# Supplementary material for: Splice-Junction-Based Mapping of Alternative Isoforms in the Human Proteome
Source: Cell Rep. Author manuscript; Available in PMC 2020 Jan 15. (PMC6961840; doi:10.1016/j.celrep.2019.11.026)
Supplement: 2 [file NIHMS1546469-supplement-2.zip › DF1/PXD006675/VentricularSeptum/VentricularSeptum_27_RILPL1_ASSDAAESALEK.pdf]

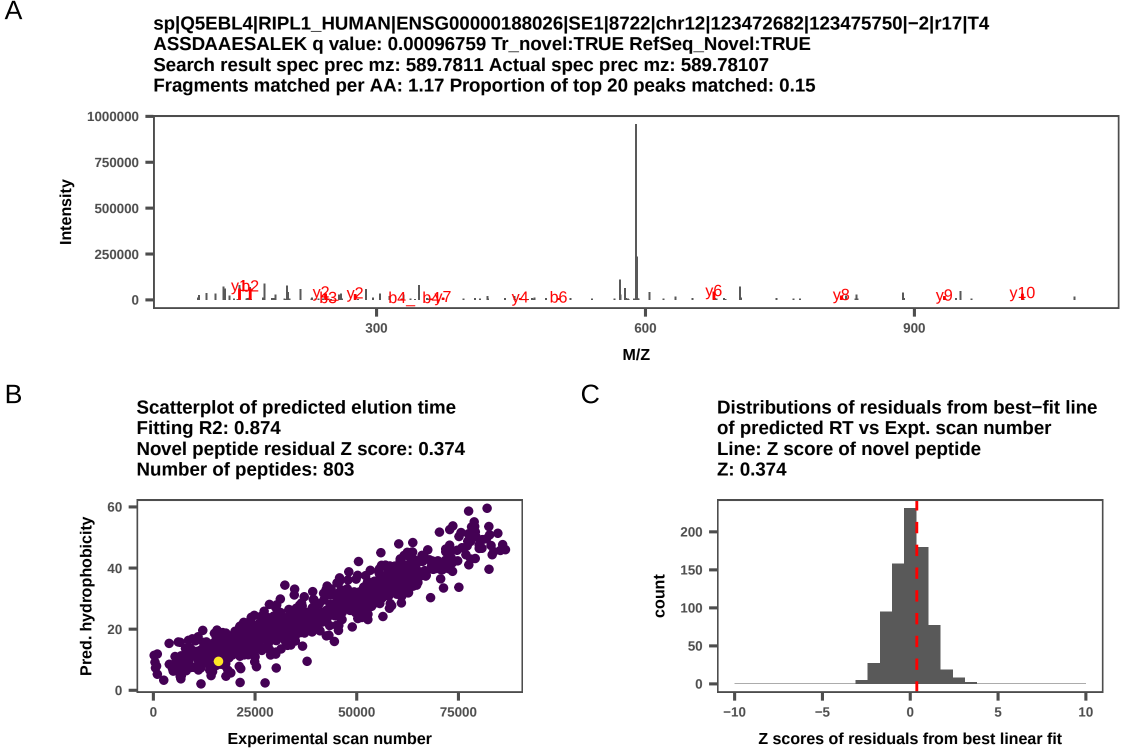
A
sp|Q5EBL4|RIPL1_HUMAN|ENSG00000188026|SE1|8722|chr12|123472682|123475750|−2|r17|T4
ASSDAAESALEK q value: 0.00096759 Tr_novel:TRUE RefSeq_Novel:TRUE
Search result spec prec mz: 589.7811 Actual spec prec mz: 589.78107
Fragments matched per AA: 1.17 Proportion of top 20 peaks matched: 0.15
Intensity
1000000
750000
500000
250000
0
y4
b6
y6
y8
y9
y10
300
600
900
M/Z
B
Scatterplot of predicted elution time
Fitting R2: 0.874
Novel peptide residual Z score: 0.374
Number of peptides: 803
Pred. hydrophobicity
60
40
20
0
0
25000
50000
75000
Experimental scan number
C
Distributions of residuals from best−fit line
of predicted RT vs Expt. scan number
Line: Z score of novel peptide
Z: 0.374
count
200
150
100
50
0
−10
−5
0
5
10
Z scores of residuals from best linear fit
